# Supplementary material for: A set of multi-entry identification keys to African frugivorous flies (Diptera, Tephritidae)
Source: Zookeys. 2014 Jul 24;(428):97–108. doi: 10.3897/zookeys.428.7366 (PMC4143993; doi:10.3897/zookeys.428.7366)
Supplement: Supplementary material 4 — Key to Capparimyia [file zookeys-428-097-s004.zip › SF4_ZooKeys_key to Capparimyia/lucid_player/help/help/collapsing.htm]

Collapsing and expanding tree items 


# Collapsing and expanding tree items

In Lucid3 keys, features and entities can be arranged in hierarchical
trees. Where this is the case, nodes of the tree are opened and closed
using buttons to the left of each node:

Alternatively, the Collapse All and Open All buttons on the toolbar
   will
collapse or open all nodes.

Note that at start-up the *Features Remaining* tree
will be collapsed to its root branches.
